# P-1345. Machine Learning-driven Insights into Mortality Risk Among MRSA Bacteremia Patients on Pharmacist-led Vancomycin Dosing

**DOI:** 10.1093/ofid/ofaf695.1533

**Published:** 2026-01-11

**Authors:** Tommy Hing-cheung Tang, Kitty Tsz-ming Ng, Qianna U qing Chung, Helen Shuk-ying Chan, Sally Lok-ting Law, Vivien Wing-man Ho, Jacky Zhen-hao Goh, Man-yee Chu, Ruby Tsz-shan Kwong, Kwok-wai Lam, Wan-man Ting, Joe Lok-fung Tung, Sai-kwong Yung, Tak-chiu Wu

**Affiliations:** Department of Medicine, Queen Elizabeth Hospital, Hong Kong SAR, China, Hong Kong SAR, China, Hong Kong; Department of Pharmacy, Queen Elizabeth Hospital, Hong Kong SAR, China, Hong Kong SAR, China, Not Applicable, Hong Kong; Queen Elizabeth Hospital, Hong Kong, Hong Kong SAR, China, Hong Kong; Department of Medicine, Queen Elizabeth Hospital, Hong Kong SAR, China, Hong Kong SAR, China, Hong Kong; Department of Pharmacy, Queen Elizabeth Hospital, Hong Kong SAR, China, Hong Kong SAR, China, Not Applicable, Hong Kong; Department of Pharmacy, Queen Elizabeth Hospital, Hong Kong SAR, China, Hong Kong SAR, China, Not Applicable, Hong Kong; Department of Pharmacy, Queen Elizabeth Hospital, Hong Kong SAR, China, Hong Kong SAR, China, Not Applicable, Hong Kong; Department of Medicine, Queen Elizabeth Hospital, Hong Kong SAR, China, Hong Kong SAR, China, Hong Kong; Department of Medicine, Queen Elizabeth Hospital, Hong Kong SAR, China, Hong Kong SAR, China, Hong Kong; Department of Medicine, Queen Elizabeth Hospital, Hong Kong SAR, China, Hong Kong SAR, China, Hong Kong; Department of Medicine, Queen Elizabeth Hospital, Hong Kong SAR, China, Hong Kong SAR, China, Hong Kong; Department of Medicine, Queen Elizabeth Hospital, Hong Kong SAR, China, Hong Kong SAR, China, Hong Kong; Department of Medicine, Queen Elizabeth Hospital, Hong Kong SAR, China, Hong Kong SAR, China, Hong Kong; Department of Medicine, Queen Elizabeth Hospital, Hong Kong SAR, China, Hong Kong SAR, China, Hong Kong

## Abstract

**Background:**

Vancomycin dosing strategies guided by therapeutic drug monitoring (TDM) data are advocated in serious methicillin-resistant *Staphylococcus aureus* (MRSA) infections. Pharmacist-led vancomycin dosing was started in 2020 at the Queen Elizabeth Hospital (QEH), Hong Kong SAR, China. Doses were adjusted by either area under curve/minimal inhibitory concentration (AUC/MIC)-guided or trough-guided monitoring, depending on patient characteristics. This study analyzed patients with MRSA bacteremia and factors linked to mortality. It was approved by the Hospital Authority Central Institutional Review Board (CIRB-2025-019-1).

**Table 1.**
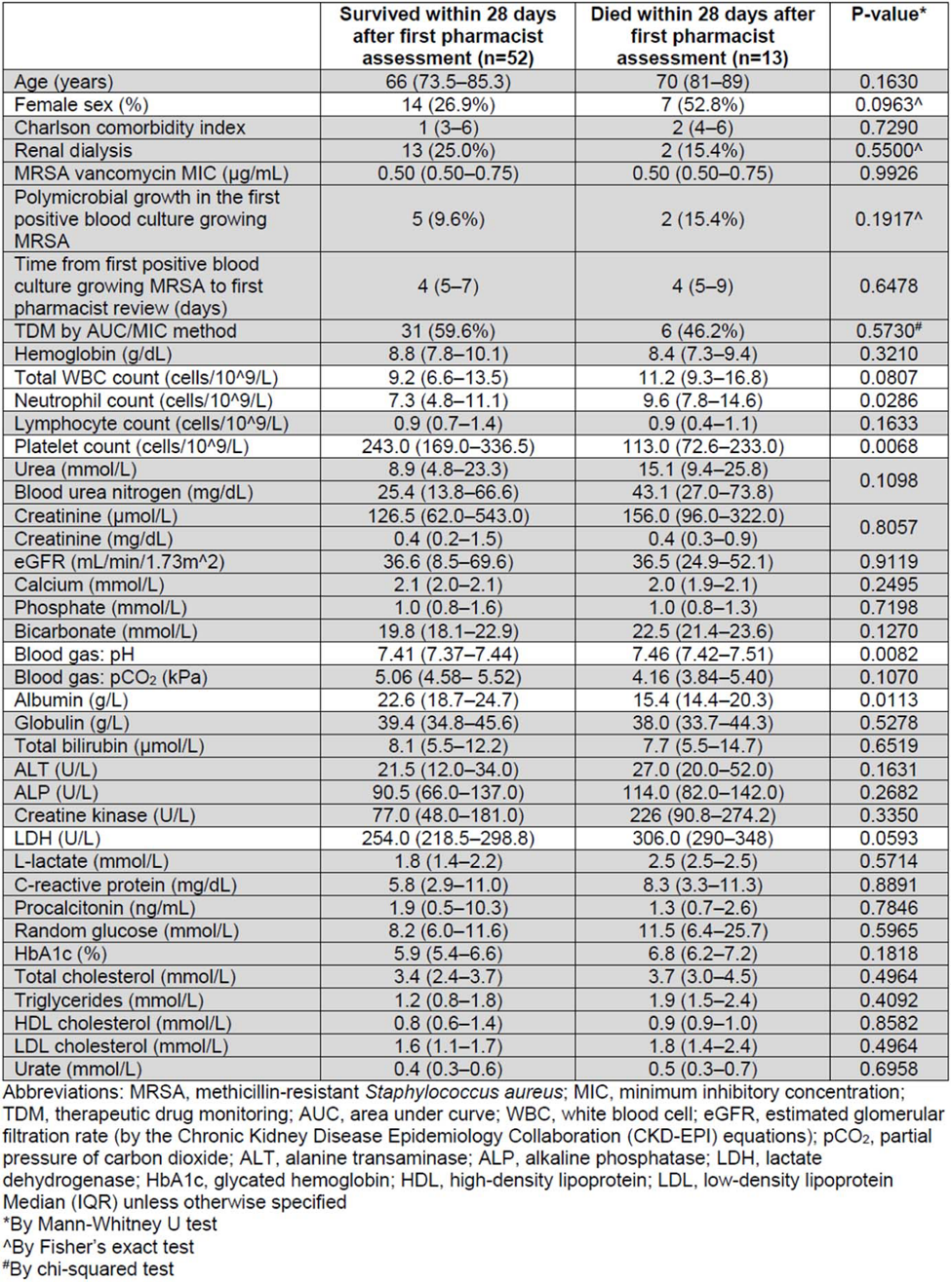
Baseline characteristics of patients with MRSA bacteremia required vancomycin therapeutic drug monitoring: at/before the first pharmacist assessment

**Methods:**

We identified adults admitted to the medical department of QEH and had at least one pharmacist-led dosing review for MRSA bacteremia. Thirty-eight baseline characteristics were compared between patients who survived or died within 28 days of the first pharmacist assessment. From these variables we predicted mortality by selecting potential predictors via a hybrid approach, combining statistical tests (p < 0.1) with feature importance from XGBoost machine learning algorithm.

**Results:**

From 2020 to November 2024, 65 patients were identified. Twenty-one (32.3%) were female and 15 (23.1%) required renal dialysis. Vancomycin MIC was less than 1 μg/mL in 96.9% of the 65 first MRSA blood isolates. Dosing was reviewed at a median of 5 times (IQR 3–8). Thirteen (20%) died within 28 days after the first pharmacist assessment. Table 1 shows the details. Six variables (sex, white blood cell, neutrophil, platelet, albumin, lactate dehydrogenase, and blood gas pH) were selected (p < 0.1). A XGBoost model predicted 28-day mortality in five-fold cross-validation and yielded a training error of 6.5% (93.5% accuracy), a test error of 21.5% (78.5% accuracy) and an AUC of 0.73. Platelet, albumin, and blood gas pH were the 3 most important features which contributed 46.1%, 39.7%, and 14.1% to predications respectively. Cut-offs to predict mortality were 199 cells/10^9/L (platelet), 19 g/L (albumin), and 7.44 (blood gas pH).

**Conclusion:**

Despite individualized vancomycin dosing, MRSA bacteremia still carries a high mortality risk. By modeling, platelet, albumin, and blood gas pH might be important predictors in this cohort. More studies with larger sample sizes are needed.

**Disclosures:**

All Authors: No reported disclosures

